# Dependence of the damage in optical metal/dielectric coatings on the energy of ions in irradiation experiments for space qualification

**DOI:** 10.1038/s41598-021-82860-7

**Published:** 2021-02-09

**Authors:** Maria G. Pelizzo, Alain J. Corso, Giovanni Santi, René Hübner, Denis Garoli, Dominic Doyle, Philip Lubin, Alexander N. Cohen, Jacob Erlikhman, Giulio Favaro, Marco Bazzan, Jon Drobny, Davide Curreli, Maxim Umansky

**Affiliations:** 1 Consiglio Nazionale delle Ricerche - Istituto di Fotonica e Nanotecnologie (CNR-IFN), Via Trasea, 7, 35131 Padua, Italy; 2 Centro di Ateneo di Studi e Attività Spaziali (CISAS), Università di Padova, Via Venezia, 15, 35131 Padua, Italy; 3 Helmholtz-Zentrum Dresden-Rossendorf Institute of Ion Beam Physics and Materials Research Ion Beam Center, Bautzner Landstr. 400, 01328 Dresden, Germany; 4 Istituto Italiano di Tecnologia, Via Morego, 30, 16163 Genoa, Italy; 5 Faculty of Science and Technology Free University of Bozen, Piazza Università 5, 39100 Bolzano, Italy; 6 ESTEC-European Space Agency, Keplerlaan 1, 2200 AG Noordwijk, ZH The Netherlands; 7 Department of Physics, University of California Santa Barbara, Santa Barbara, CA 93106 USA; 8 Dipartimento di Fisica e Astronomia, Università di Padova, Via Marzolo, 8, 35131 Padua, Italy; 9 Department of Nuclear, Plasma, and Radiological Engineering, University of Illinois at Urbana Champaign, Urbana, IL 61801 USA; 10 Lawrence Livermore National Laboratory, Livermore, CA 94550 USA

**Keywords:** Astronomical instrumentation, Characterization and analytical techniques, Aerospace engineering, Surfaces, interfaces and thin films, Engineering, Materials science, Optics and photonics

## Abstract

Terrestrial accelerator facilities can generate ion beams which enable the testing of the resistance of materials and thin film coatings to be used in the space environment. In this work, a $\hbox {TiO}_2$/Al bi-layer coating has been irradiated with a $\hbox {He}^+$ beam at three different energies. The same flux and dose have been used in order to investigate the damage dependence on the energy. The energies were selected to be in the range 4–100 keV, in order to consider those associated to the quiet solar wind and to the particles present in the near-Earth space environment. The optical, morphological and structural modifications have been investigated by using various techniques. Surprisingly, the most damaged sample is the one irradiated at the intermediate energy, which, on the other hand, corresponds to the case in which the interface between the two layers is more stressed. Results demonstrate that ion energies for irradiation tests must be carefully selected to properly qualify space components.

## Introduction

The study of the behavior of optical materials and coatings in a space environment is pivotal for the realization and optimization of scientific instrumentation, navigation sensors, detectors, and solar panels. With the advent of new space missions operating in particularly hostile environments, such as those in low-perihelion solar orbits and those studying the Jovian magnetosphere, the need to understand the effects of the irradiation of charged particles such as electrons, protons, and He ions has emerged^1^. In fact, radiation-induced modification and/or degradation of optical materials and thin films affects the response and the efficiency of the instrumentation, and, in case of fatal damage, determines its failure^2^.

The characteristic energy of charged particles is associated with their source and dynamics. MeV protons and electrons are abundant, for example in low-earth-orbit, as they are trapped in the Van Allen belts^3^. Nevertheless, due to their limited thickness (between few and hundreds of nanometers), thin film coatings are mostly affected by keV ions, as these implant within the material, changing their optical and structural properties. Ions in the keV energy range are constituents of the solar wind, an outflow of plasma, originating from the solar corona and expanding outwards into the interplanetary region. Among the different types of solar wind, which are characterized by different velocities and durations of propagation, the quiet solar wind is the one that carries particles with the lowest kinetic energy, typically 1 keV for protons and 4 keV for $\alpha$-particles. Distinct from solar wind components associated with eruption phenomena such as coronal mass ejections and solar flares, the quiet solar wind flows constantly, and, for this reason, it is considered a long-term source of degradation of space components^2,4^. Low-energy particles have also been demonstrated to be present in planetary atmospheres, including terrestrial orbits^5^.

The study of the changes induced in optical coatings and substrates by charged particles is carried out using terrestrial accelerator facilities, often underestimating the importance of appropriately selecting and controlling the irradiation parameters used. For example, the high particle flux rates used to reach the expected doses in a reasonable amount of time can induce thermal processes not present in space, where the exposure often lasts years. The ground validation of optical components is usually performed by evaluating the effect of the total ionizing dose (TID), which is the dose quantity that measures the energy deposited in matter by ionizing radiation per unit mass. By definition, TID is an integral dose, and therefore, the energy spectrum of particles can be selected arbitrarily. Generally, the selection of the ion energies is made for practical reasons, such as those related to the availability of accelerators. Indeed, parameter selection should be driven primarily by the study of the potential physical effects, since they are finely energy-dependent, as will be demonstrated in the present work.

Ions with energies in the range of a few keV up to hundreds of keV implant in the coatings, and the profile of the implantation distribution is dependent on the thin film density. Previous studies of low-energy He ion irradiation in metals, such as W, Au, and Cu, demonstrate the formation of bubbles in the film for doses of the order of $10^{17}$ $\hbox {cm}^{-2}$^6–8^ associated with a degradation of the optical performance^7^, while at doses of $10^{16}$ $\hbox {cm}^{-2}$, a faint dislocation band starts forming^8^, with preservation of the optical performance in the visible spectral range, but not in the ultraviolet^9^. A sponge-like morphology was observed in the case of gold due to bubble formation, which releases He at the film surface^7,10^. The diameter of the bubbles was shown to increase with the dose, such that for values of the order of $5\times 10^{17}$ $\hbox {cm}^{-2}$, formation of large blisters is observed. From experiments related to nuclear-reactor-associated technologies, He is known to agglomerate into bubbles when implanted not only in metals, but also in semiconductors^11^. Nano-bubbles with diameters ranging from sub-1 to 3 nm form in Si, which within the ion propagation path becomes completely amorphous^8^. Nano-bubbles are present only in the amorphous Si band due to the high vacancy-enhanced diffusion of He in crystalline Si and low mobility of He in amorphous Si. Again, both the density and the diameters of the nano-bubbles increase with the dose, reaching values up to 30 nm for doses of the order of $5\times 10^{17}$ $\hbox {cm}^{-2}$.

In the case of coatings based on dielectric materials, only few experiments of low-energy ion irradiation on optical coatings have been systematically performed and published. They are generally carried out with doses of about $10^{17}$ $\hbox {cm}^{-2}$. More often, dielectric films and stacks together with glass materials are qualified using $\gamma$-ray irradiation experiments^12–14^. In these cases, the high energy is uniformly released along the whole thickness of the sample, so that the outcomes of such experiments cannot be directly interpreted as representative of an ion irradiation test. In fact, in this last case, low-energy particles implant in the material following a precise dose-depth profile. By adopting an equivalent aluminum shielding thickness model, it is anyway possible to compute the ionizing dose deposited through the thickness of an Al film and then to derive the deposited dose-depth profile for any material by using a scaling process^15^. Experiments with low-energy protons and doses between $10^{12}$ and $10^{15}$ $\hbox {cm}^{-2}$ were carried out^16,17^. Changes in the optical performance of various oxides were observed and modelled in terms of refraction index changes. This approach was also used in the case of testing glass materials^18^. Various experiments of $\hbox {He}^{+}$ irradiation on thin film metal multilayers used in nuclear physics applications were carried out^19–21^. The analysis of the irradiated samples with doses of the order of $10^{17}$ $\hbox {cm}^{-2}$ shows bubble formation and accumulation at interfaces in multilayers with layer thicknesses above 5 nm. However, the analysis of the irradiated samples focuses on the microstructure and mechanical properties to show that irradiation improves the hardness of the samples, while the present work focuses on optical property changes. A proton irradiation test on a bi-layer optical coating is reported in^22^. In particular, an $\hbox {SiO}_2$/Al bi-layer was irradiated with a 200 keV beam up to doses of the order $10^{17}$ $\hbox {cm}^{-2}$. The purpose of the work was to study the optical degradation of the Al film. For this reason, the ion energy was selected in order to guarantee the implantation in the metals. The present work is aimed at studying the effects of irradiation as a function of the ion energy, complementing the study reported in^22^. The higher density of the $\hbox {TiO}_2$ should provide a greater obstacle to the penetration of ions into the structure than $\hbox {SiO}_2$. E-beam evaporated $\hbox {TiO}_2$/Al bi-layers for space applications have recently been studied to assess their mechanical and thermal radiation properties^23^. In the present work, a bi-layer $\hbox {TiO}_2$/Al coating was irradiated with three different ion energies in order to characterize the effect in the metal itself, in the dielectric protective layer and at the interface between the two. The $\hbox {TiO}_2$/Al bi-layer was irradiated with a $\hbox {He}^+$ beam of 4, 16, and 100 keV using the same flux and dose. In this way, the energy-dependent damage in the coating can be investigated. The energies were selected considering that the ions of the quiet solar wind carry the lowest kinetic energy^24^, while particles in the range of 100 keV can be found in the near-Earth space environment^25^. In particular, 4 keV $\hbox {He}^+$ are associated with quiet solar wind particles in the ecliptic plane, while 16 keV ions are abundant in polar solar orbits. The dose reached with the present experiment is $4\times 10^{17}$ $\hbox {cm}^{-2}$, in order to explore the regime in which blistering occurs.

## Materials and methods

$\hbox {TiO}_2$(90 nm)/Al(340 nm) thin films were deposited using electron-beam evaporation on Si substrate, by including an adhesion layer of a few nm of chromium. The Si substrates are Czochralski-grown p-type polished wafers with a thickness of 650–700 $\upmu$m. All materials were deposited by electron-beam evaporation with a base vacuum of 10$^{-6}$ mbar (i.e. 10$^{-4}$ Pa) and at a temperature of 27 $^\circ$C. The layers’ thicknesses were in-line monitored with a quartz micro-balance. The Al source consisted of 99.99%-purity pellets placed in an inter-metallic crucible, whereas the $\hbox {TiO}_2$ source was composed of 99.9%-purity pellets placed in a FABMATE(R) crucible (i.e. high-strength, pure graphite). Three different specimens of the deposited films were irradiated with $\hbox {He}^+$ ions at different energies, as summarized in Table 1, while some remaining specimens were stored in a regular environment and kept as reference samples.

**Table 1:** Summary of the irradiation parameters considered and used in the tests.

Session	Ion species	Energy (keV)	Flux ($\hbox {cm}^{-2}\hbox {s}^{-1}$)	Dose ($\hbox {cm}^{-2}$)
1	$\hbox {He}^+$	4	$1.6\times 10^{13}$	$4.0\times 10^{17}$
2	$\hbox {He}^+$	16	$1.6\times 10^{13}$	$4.0\times 10^{17}$
3	$\hbox {He}^+$	100	$1.6\times 10^{13}$	$4.0\times 10^{17}$

**Figure 1:**
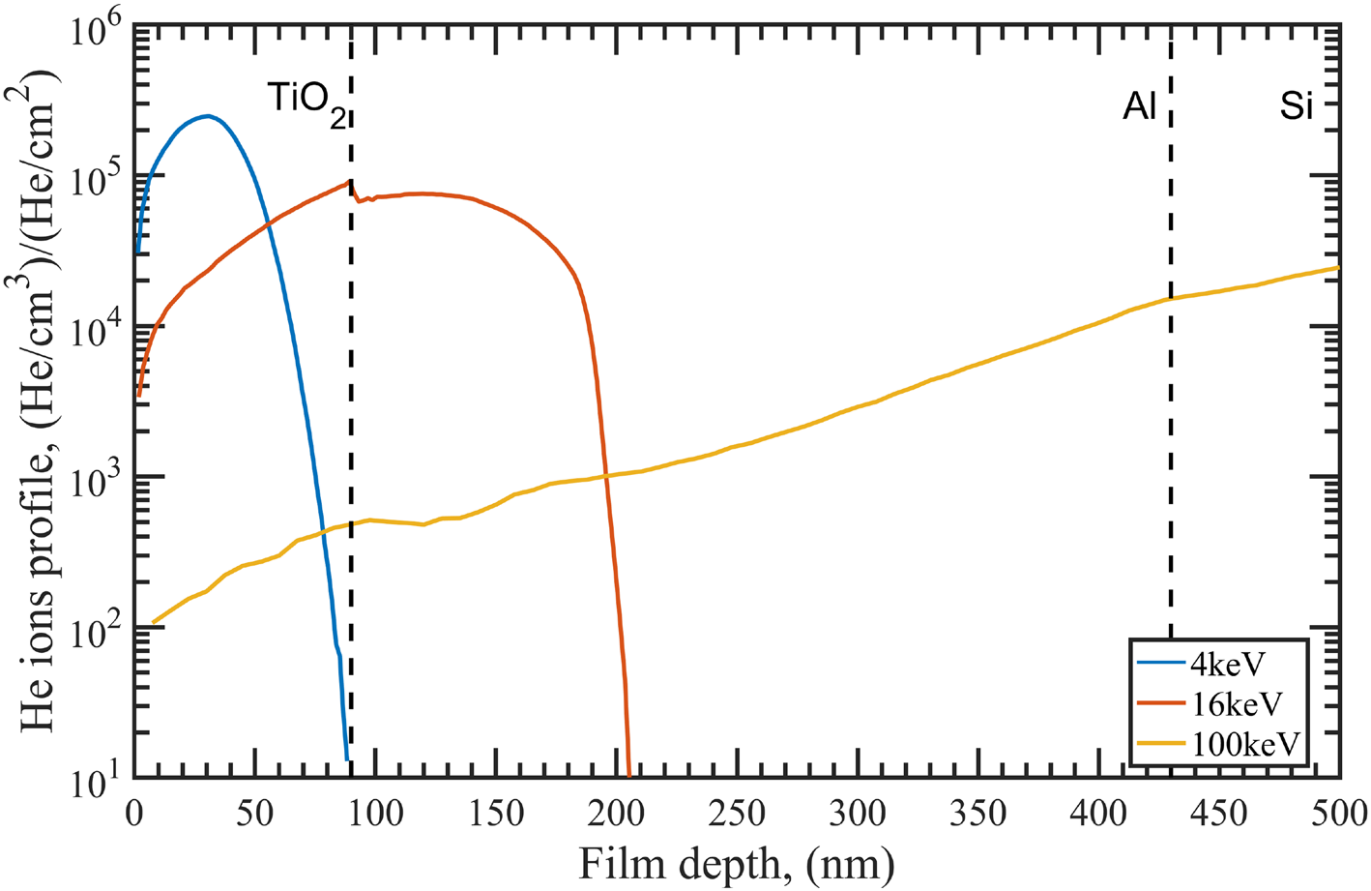
Stopping range of $\hbox {He}^+$ ions determined by simulations of collision dynamics with the TRIM/SRIM software for the energies used in the experiments.

The Al and $\hbox {TiO}_2$ layers’ thicknesses were optimized in order to have three different situations, which correspond to the three different energies: (1) in the 4 keV case, the ions are implanted in the dielecric protective-layer; (2) in the case of 16 keV irradiation energy, the peak of the implantation profile is placed at the interface between Al and $\hbox {TiO}_2$; (3) in the 100 keV case, the $\hbox {He}^+$ penetrate through the entire depth of the coating and reach the substrate. The stopping range of the ions was determined by simulating the collision dynamics by means of the TRIM/SRIM software^26^. The ion penetration depth versus energy is shown in Fig. 1. Due to the differing densities between the oxide and the Al layer, a correspondingly abrupt change is observed in the profile at the interface.

Irradiation experiments at 4 keV and 16 keV were performed by using the Danfysik A/S 40 kV ion implanter at the Ion Beam Center (IBC) of the Helmholtz–Zentrum Dresden–Rossendorf (HZDR) in Germany. The ion energies were achieved by setting an extraction potential at − 20 kV and an acceleration potential of either 16 kV or 4 kV in order to obtain the target ions energy of 4 keV or 16 keV, respectively. The irradiation at 100 keV was performed by using the Danfysik A/S 200 kV ion implanter at the IBC of the HZDR with an extraction potential set at 30 kV and an accelleration potential of 70 kV. For all the implantation sessions, the ion beam was focused with a spot size of $\sim$ 5 mm and electrostatically raster-scanned at a frequency of $\sim$ 1 kHz by using horizontal and vertical pairs of deflection plates. Homogeneity was monitored by comparing the measured ion beam currents in the Faraday cups at the four corners, which must be equal. During the implantation, the experimental chamber base vacuum was about $10^{-7}$ mbar (i.e. 10$^{-5}$ Pa), and the samples were placed at 7$^\circ$ off normal incidence in order to prevent possible channeling effects. The ion current was integrated over time in order to control the total dose, while the flux was kept fixed at the values reported in Table 2.

The morphological, structural and optical properties were characterized for both the irradiated and non-irradiated samples. The surface morphology was characterized by using an XE-70 Park System Atomic Force Microscope (AFM) operated in non-contact mode. Moreover, high-resolution field-emission Scanning Electron Microscopy (SEM) was performed by using an FEI HeliosNanolab650 system operated at an accelerating voltage of 10 kV. Both top- and tilted-view micrographs were acquired. The spectral reflectance of the samples was measured in the 250–900 nm wavelength range by using a Cary 5000 double-grating spectrophotometer. The measurements were performed by employing a VW-scheme which allows measuring the absolute reflectance of the samples with an incidence angle of 7$^{\circ }$ and an accuracy better than $2\%$.

The samples’ scattering performance was evaluated by estimating the Total Integrated Scattering (TIS), defined as

1 $$\begin{aligned} {\mathrm {TIS}}=\frac{R_{d}}{R_{d}+{R_s}}, \end{aligned}$$

where $R_s$ is the specular reflectance and $R_d$ is the diffuse reflectance. Both $R_s$ and $R_d$ were measured in the 350–850 nm spectral range by using the UV–Vis-NIR Internal Diffuse Reflectance Accessory (IDRA) supplied with the Cary 5000 spectrophotometer. The accessory consists of an integrating sphere, in which the sample can be mounted into two different configurations: in the first configuration, the sample is placed with an incidence angle of 3.5$^{\circ }$ with respect to the probing beam, allowing the measurement of the totally reflected (i.e. specular and diffuse, $R_{d}+{R_s}$) light; in the second configuration, the sample is placed exactly at normal incidence, allowing the back-reflection of the probing beam and the measurement of the diffuse component only. The accuracy of the measured TIS is estimated to be about $4\%$.

In addition, spectroscopic ellipsometry was performed by a VASE Spectroscopic Ellipsometer (J.A. Woollam). The goniometer-controlled optical bench was set for three different angles of incidence on the sample ($45^\circ$, $55^\circ$, $65^\circ$), and the ellipsometric data were recorded in the rotating polarizer analysis setup in the range 400–900 nm, with a step size of 10 nm. The output of the ellipsometric analysis is the ratio $\rho$ of the p-polarized and s-polarized complex Fresnel reflection coefficients $r_p$ and $r_s$, expressed in terms of the ellipsometric angles *Psi* and $\Delta$:

2 $$\begin{aligned} \frac{r_p}{r_s}=\tan \Psi \cdot e^{i \cdot \Delta } \end{aligned}$$

Afterwards, the data were analyzed with the WVASE32 software (J.A. Woollam), provided by the manufacturer. Samples are usually modeled as a stack of *n* layers, depending on sample complexity, each one characterized by its complex dielectric function and its effective thickness. Thus, experimental data were fitted until the best agreement with the model was achieved, and an estimation of the optical constants and thickness of each layer was eventually provided.

The structural properties of the He-implanted films were investigated by cross-sectional bright-field Transmission Electron Microscopy (TEM). These analyses were performed using an image-$\hbox {C}_s$-corrected Titan 80-300 microscope (FEI) operated at an accelerating voltage of 300 kV. Classical TEM cross-sections of the $\hbox {He}^+$ broad-beam irradiated $\hbox {TiO}_2$/Al bi-layer samples glued together in face-to-face geometry using G2 epoxy glue (Gatan) were prepared by sawing (Wire Saw WS 22, IBS GmbH), grinding (MetaServ 250, Bühler), polishing (Minimet 1000, Bühler), dimpling (Dimple Grinder 656, Gatan), and last $\hbox {Ar}^+$ ion milling (Precision Ion Polishing System PIPS 691, Gatan).

The crystalline state of the coating before and after the implantation process was investigated by grazing incidence X-ray diffraction, performed by keeping the incidence angle ($\omega$) of the X-ray beam fixed at $1^{\circ }$, $3^{\circ }$ and $5^{\circ }$ with respect to the sample surface and scanning from $20^{\circ }$ to $120^{\circ }$ with the detector angle ($2\theta$). The data were acquired on a Philips MRD diffractometer operated at 40 kV, 40 mA using Cu K$\alpha$ radiation ($\lambda =1.54056 {\AA }$). The primary optics consist of a parabolic multilayer mirror collimating and partially removing the contribution of other X-ray lines in the primary beam. Both the sample and the detector (a Xe proportional counter) are mounted on two co-axial high-precision goniometers (accuracy of $0.0005^{\circ }$). Analysis of the diffractograms was carried out by performing a Rietveld^27^ refinement with the MAUD software^28^.

## Results and discussion

### Reflectance performance

**Figure 2:**
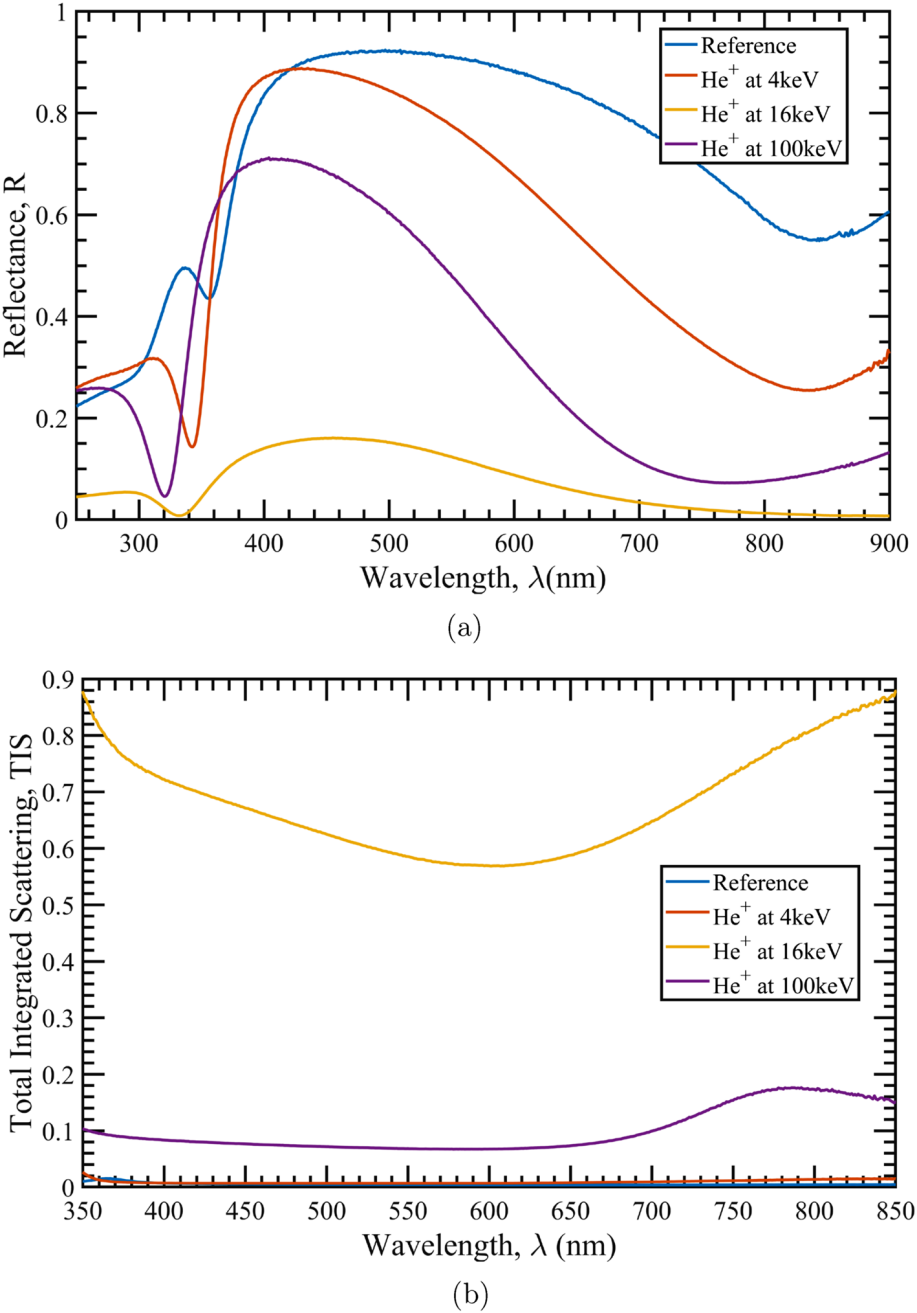
(**a**): Spectral reflectance of the $\hbox {TiO}_2$/Al samples irradiated at different energies. (**b**): Total integrated scattering (TIS) measured for indicated the samples.

The spectral specular reflectance of the irradiated samples together with the reference (not irradiated) is reported in Fig. 2a.

All the irradiated samples show a degradation in performance, which surprisingly does not increase as expected as energy increases. In fact, the sample which exhibits the most radiation–induced damage is the one which was irradiated at 16 keV. This can be partially explained by observing the implantation profiles reported in Fig. 1. In the case of 4 keV irradiation, the implanted ions are completely confined in the $\hbox {TiO}_2$ capping-layer protecting the metallic film underneath. Thus, the irradiation effects occurring in the dielectric layer (i.e. refractive index changes and little scattering due to bubble formation) must be the principle cause of the observed degradation of the optical performance, even though the Al layer still maintains a good spectral reflectance. While the observed shift in the reflectance peak around 350 nm suggests changes in the dielectric constant and thickness of the capping-layer, the presence of the Al interband transition feature close to 800 nm confirms that the Al layer is not significantly damaged. Conversely, in the case of the 16 keV-irradiated sample, the reflectance curve is seriously modified. As shown in Fig. 1, in this case, the implantation profile peak falls exactly at the interface between the capping-layer and the metal layer; in particular, from Fig. 1 it can be seen that the upper 50 nm of Al are affected by a large amount of He inclusion, with a volume fraction (i.e. the ratio between the number of He atoms and Al atoms) of about 0.5, which deeply changes the metal’s morphological, structural and optical properties. The degradation is particularly dramatic because the reflectance is predominantly determined by the degradation of the interface between the $\hbox {TiO}_2$ capping-layer and the first few tens of nm of the Al layer; in fact, starting from the extinction coefficient *k*, the penetration depth $\gamma _p$ estimated for aluminum is $< 7.5$ nm in the visible spectral range, so that all the changes in the Al layer occurring deeper than $\simeq 2\gamma _p=15$ nm do not significantly affect the reflectance performance. Finally, considering the more uniform ion implantation curve at 100 keV, with a broad peak within the substrate, the ions are deposited throughout the entire coating stack, but with low volume fractions (i.e. $\simeq$ 0.01 or less). Both the whole capping-layer and the metal portion interacting with the light are completely affected by the high-energy ion implantation, with a final degradation of the reflectance curve larger than in the case of 4 keV, but less than in the case of 16 keV. Moreover, the shift of the Al interband transition peak to lower wavelengths suggests an effective modification of the metallic layer optical performance. The previous analysis is further strengthened by the TIS curves reported in Fig. 2b. As expected, the scattering component in the reference sample is small. By considering a Gaussian surface height distribution function, a rough estimation of the RMS surface roughness can be performed for samples having low scattering performance^29^. In the case of the reference sample, a value of 3.2 nm was estimated, which is compatible with the AFM measurements reported in “Morphological analysis” section. For the 4 keV-irradiated sample, the scattering contribution is still small, although slightly higher than that for the reference sample. The RMS roughness estimate is 4.7 nm, an underestimated value with respect to that one retrieved by AFM analysis. The TIS curve for the 16 keV-irradiated sample reveals a very high level of scattering, confirming that light-diffusion is among the dominant mechanisms that reduce the specular reflectance of the sample. Although the evaluation of the RMS roughness via TIS for samples with such high light diffusion becomes speculative, interesting information on the status of the surface can be obtained. The average RMS roughness gives a value of about 52 nm, suggesting that a huge part of the $\hbox {TiO}_2$ capping-layer undergoes a dramatic morphology change. The 100 keV-irradiated sample shows scattering as well, but much less than in the 16 keV case. The RMS roughness estimation for this sample is about 16 nm.

A deeper investigation of the optical response dependence on the ion irradiation was performed by spectroscopic ellipsometry, which confirms the above discussion (see Supporting Material 1 for a detailed description). The ellipsometric data of the reference sample were successfully fitted by using the tabulated optical constants for both $\hbox {TiO}_2$ and Al^30,31^, considering the nominal thickness of the layers (i.e. 90 nm for $\hbox {TiO}_2$ and 340 nm for the Al layer) and the surface roughness found via TIS analysis ($R_q=3.2$ nm). For the 4 keV-irradiated sample, suitable fitting is still possible with changing only the optical constants of the $\hbox {TiO}_2$ and keeping unchanged both the thicknesses and the Al optical constants. In this case, the best fit was obtained by slightly increasing the surface roughness ($R_q \simeq 6$ nm), not so far from both the value found via TIS analysis and AFM (see “Morphological analysis” section). In contrast, in the case of 16 keV, a good fit is only possible considering a strong surface roughness ($R_q \simeq 70$ nm), a strong modification of the $\hbox {TiO}_2$ optical constants, a modest change for the Al optical constants, and an inter-layer of 150 nm between the capping-layer and the Al layer for simulating, by using an Effective Medium Approximation model, the voids due to the bubbles (see the TEM analysis reported in “Structural analysis” section). The ellipsometric measurements for the 100 keV-irradiated sample can still be fitted by using the model employed in the 4-keV case, as long as the surface roughness is increased ($R_q \simeq 18$ nm) and the optical constants of the Al layer are changed.

### Structural analysis

**Figure 3:**
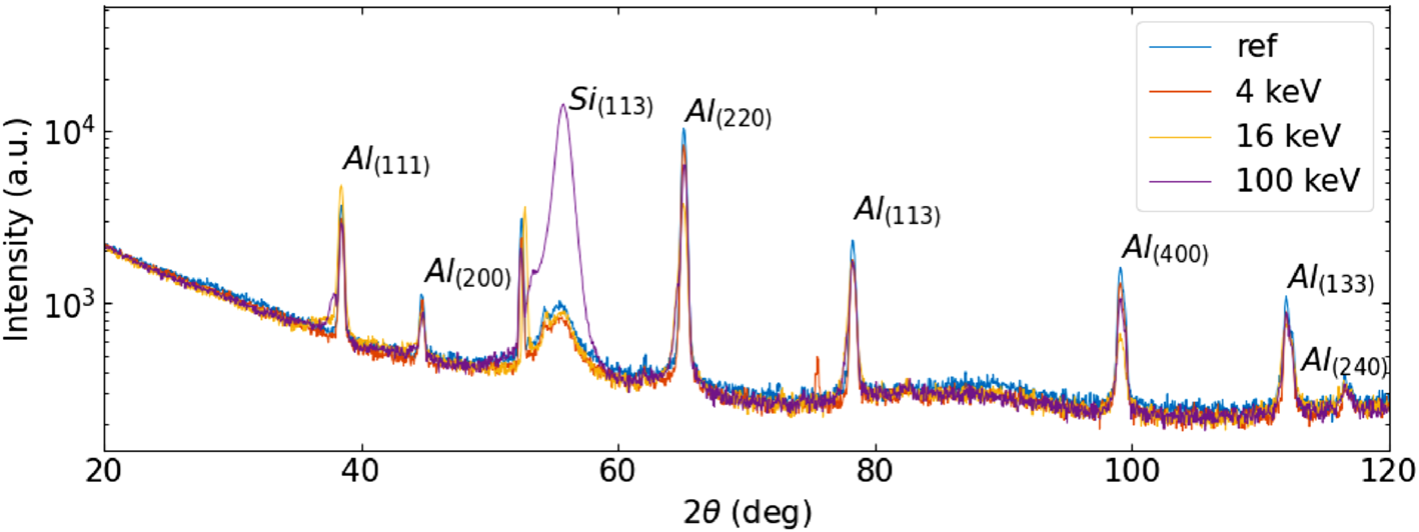
Grazing incidence XRD spectra acquired for the various $\hbox {TiO}_2$/Al samples at $1^{\circ }$ in $\omega.$

**Figure 4:**
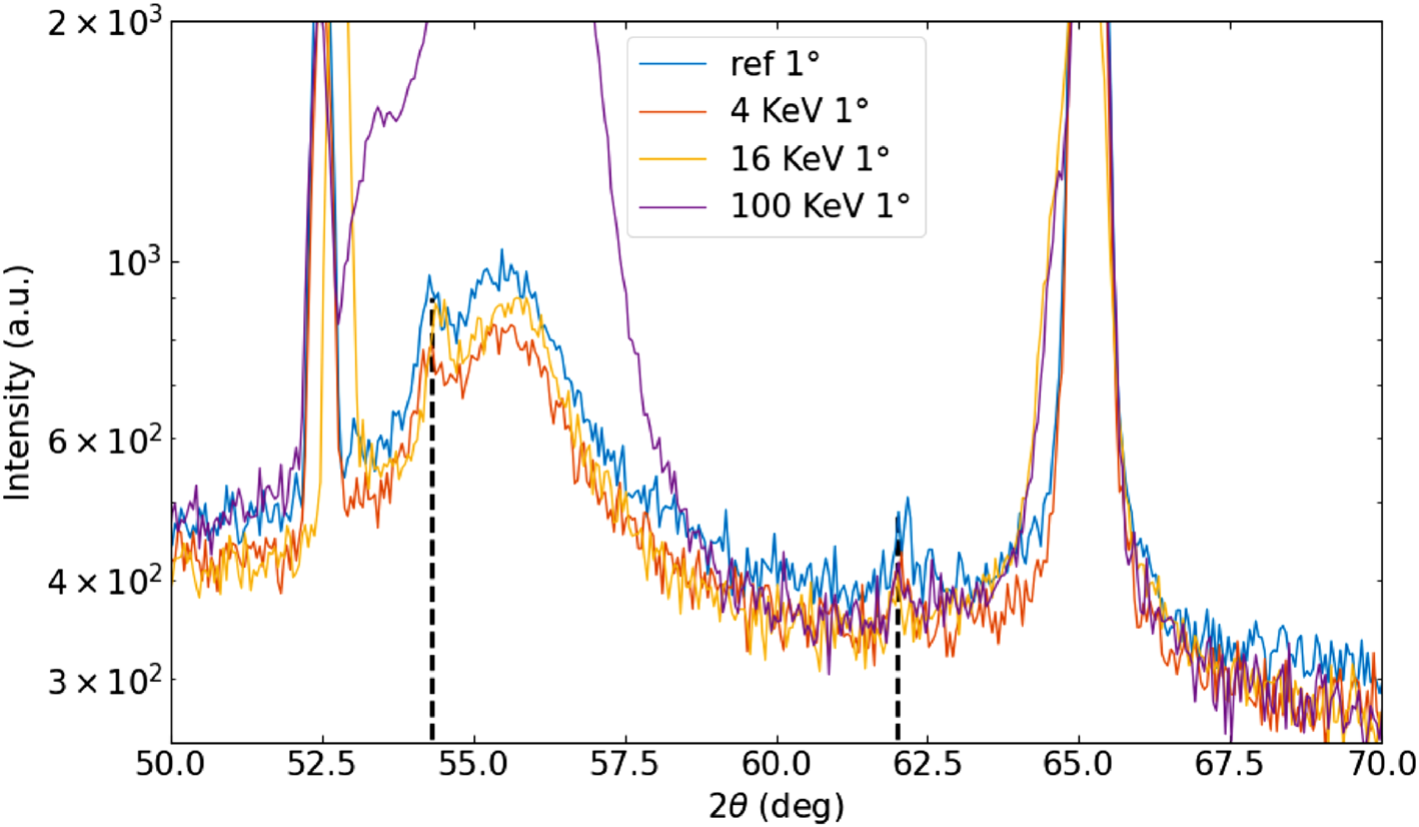
$$\hbox {TiO}_2$$ peaks candidates.

In the present experiment, the samples were prepared by an e-beam evaporation process at ambient temperature. Thin films are thus expected to be mainly amorphous^32^. This was confirmed both by Raman survey measurements, which did not show any significant peak, and by XRD analysis. A set of glancing-angle XRD measurements obtained at $\omega =1$ degree for the different samples is reported in Fig. 3. Al peaks from the bottom layer are clearly visible, indicating the presence of a polycrystalline cubic phase. In the $2\theta$ region between $50^{\circ }$ and $60^{\circ }$ other spurious structures are evident and are due to the fact that our experimental condition is close to exciting strong diffraction from the (1 1 3) planes of the single-crystalline Si substrate. In this region it is also possible to observe tiny peaks that can be related to crystalline $\hbox {TiO}_2$ in the anatase phase (see Fig. 4). This indicates that the $\hbox {TiO}_2$ layer is mainly amorphous, as expected from an e-beam evaporation process at ambient temperature and in agreement with the Raman data, but that a small crystalline fraction has developed inside the layer. Due to the very low signal-to-noise ratio, we could not study this phase in detail. Nevertheless, the observed peaks do not show any noticeable change upon the implantation dose, so we conclude that the eventual presence of a few crystallites in the $\hbox {TiO}_2$ layer is not playing a significant role.

The measurements at different incidence angles did not bring significant additional insights apart from an overall decrease of the peak intensities and a change on the relative height of the Bragg peaks, pointing out the presence of preferential orientation in the Al layer, as it is quite common with this kind of films. The data were analyzed by Rietveld fitting using the MAUD program^28^, in order to obtain the Al lattice parameter for each implantation energy. Furthermore, we estimated the Al grain size by means of the Scherrer equation, after correcting for instrumental broadening using a $\hbox {LaB}_6$ NIST standard. The results are reported in Table 2. All the samples show similar Al lattice parameters, with the exception of the 16 keV sample which shows a significant increase. At this energy, the stopping range of the He ions lies within the Al layer, thus this effect is probably due to the formation of interstitial defects expanding the lattice. On the other hand, the grain sizes for the first two implantation energies give an estimate in line with the thickness of the Al layer, indicating that the coherent diffraction volumes go through the whole size of the Al film. This can be understood as in the 4 keV case: the projected range of the He ions does not penetrate into the Al film, leaving it essentially identical to the one of the reference sample. In contrast, in the 16 keV and 100 keV implantation, the grain size appears to be significantly decreased. This can be related to the formation of extended defects due to the ion beam traversing the dielectric layers.

**Table 2:** Calculated parameters for Aluminum crystal.

	Ref.	4 keV	16 keV	100 keV
Cell parameter (Å)	4.0491(2)	4.0492(2)	4.0526(1)	4.0495(5)
Crystallite size (nm)	300(10)	280(10)	150(10)	140(10)

**Figure 5:**
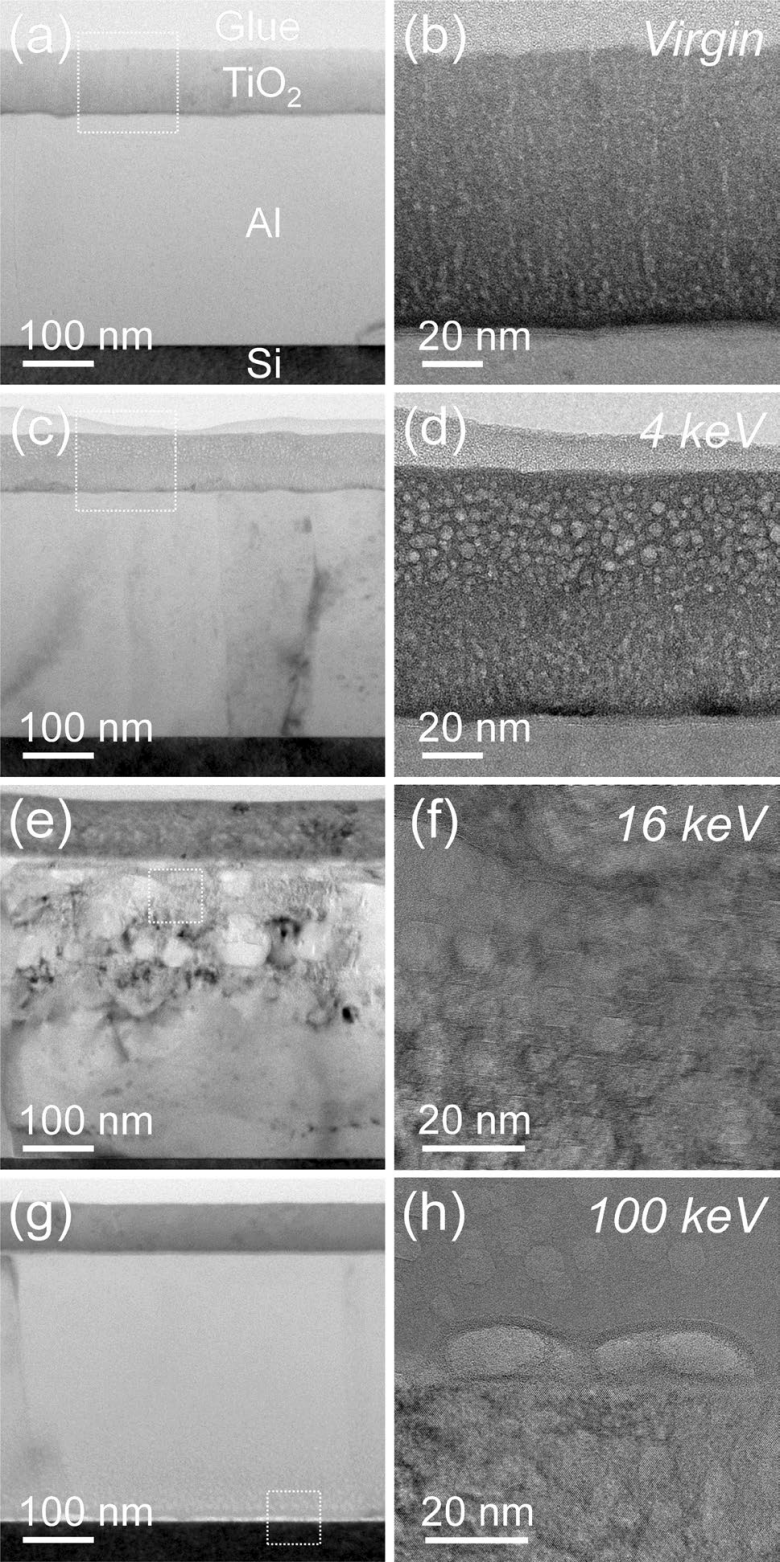
Cross-sectional TEM bright-field images (**a**, **c**, **e**, **g**) of the reference sample and the irradiated samples at 4 keV, 16 keV, and 100 keV, respectively as well as higher magnified (**b**, **d**) and high-resolution TEM images (**f**, **h**) for the areas marked in the corresponding overview images on the left side.

Figure 5a shows a representative bright-field TEM image of the non-irradiated reference sample with film thicknesses of around 330 nm for the Al and 90 nm for the $\hbox {TiO}_2$ layer. While the aluminum layer is characterized by a dense microstructure with grain sizes of the order of the film thickness, the titanium oxide layer shows a loose microstructure with elongated pores (Fig. 5b). $\hbox {He}^+$ irradiation at 4 keV does not have any apparent morphological effect on the Al layer, but leads to pronounced bubble formation in the upper half of the $\hbox {TiO}_2$ cap layer with bubble diameters of up to 10 nm (Fig. 5c, d), which, as said, cause the reflectance degradation observed.

He irradiation at 16 keV results in stronger changes of the thin film system (Fig. 5e) and, consequently, of its performance. In addition to the $\hbox {TiO}_2$ capping-layer, He bubbles are also observed in the upper half of the aluminum film. While the smaller bubbles seem to adapt the crystallographic structure of the Al film with (111) and (100) facets (Fig. 5f), larger bubbles are of more irregular shape. Due to the He bubble formation, the Al film thickness increases by at least 25%. If the He bubbles in the upper half of the Al film agglomerate and get larger, blister formation and final blister rupture can occur, as revealed by SEM and AFM analysis presented in the next section. This effect can justify the dramatic changes in the optical response of the bi-layer stack.

He irradiation at 100 keV leads to a densification of the $\hbox {TiO}_2$ capping-layer and, hence, to a reduction of the corresponding film thickness by 20$\%$ (Fig. 5g). He bubbles are predominantly present in the lower half of the Al film and the top Si substrate region (Fig. 5h). Their additional formation at the $\hbox {TiO}_2$/Al interface might lead to a reduced interfacial strength and, hence, to the blister formation; nevertheless, the impact on reflectance performance is smaller than in the case of 16 keV. It should be noted that the irradiation experiments were performed without any active control of the samples’ temperature. The high thermal stability of the Al/$\hbox {TiO}_2$ films suggests only minor effects from potential temperature gradients during the irradiation.

### Morphological analysis

**Figure 6:**
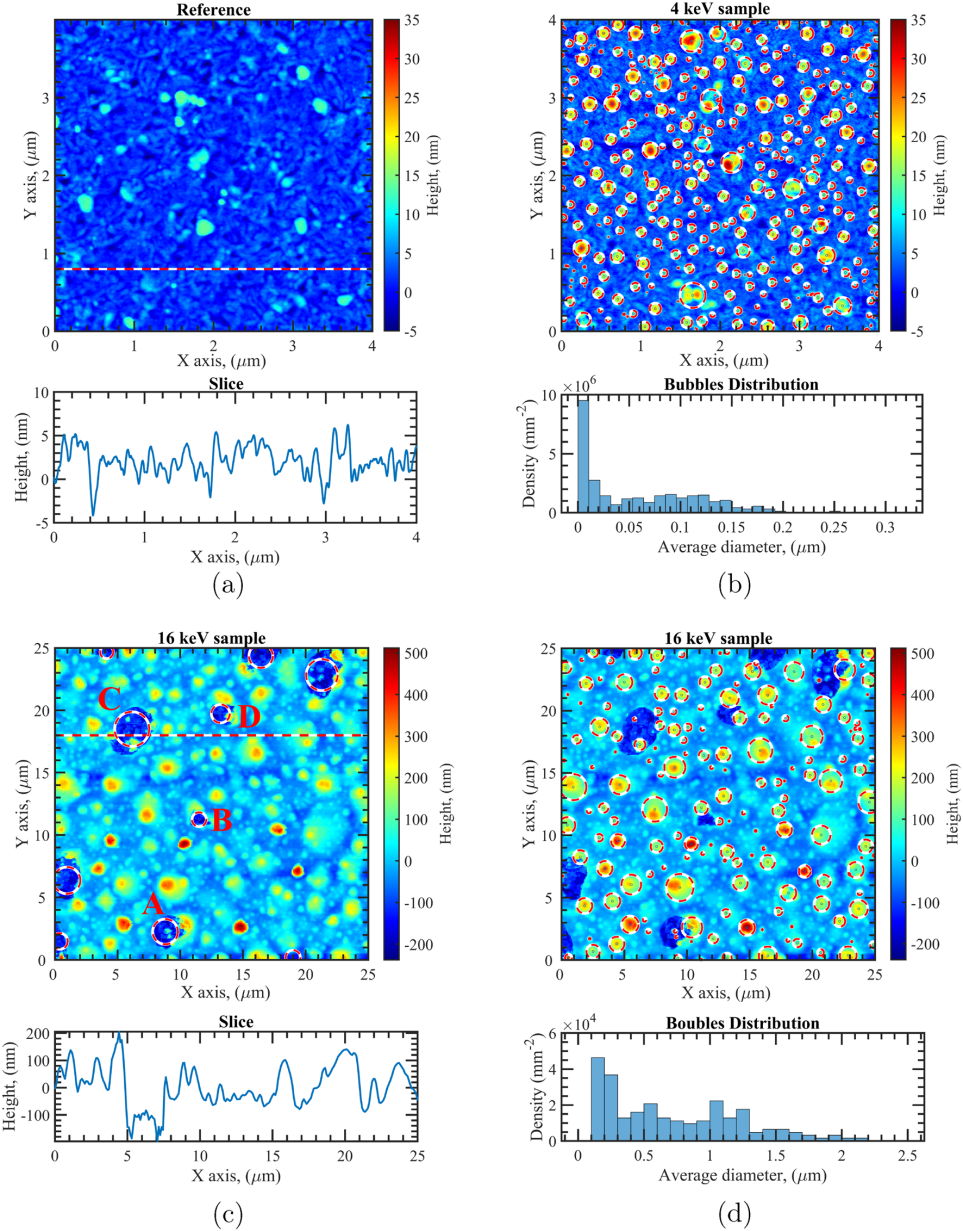
AFM images of the reference (**a**), the 4 keV-irradiated sample (**b**) and the 16 keV-irradiated sample (**c**,**d**).

AFM analysis of the reference sample reveals a quite smooth surface, characterized by a RMS roughness of $R_q=3.1$ nm and an arithmetical deviation of $R_a=2.5$ nm (Fig. 6a). The 4 keV-irradiated sample shows instead the formation of surface bubbles with diameters mainly less than 20 nm (Fig. 6b). The sample undergoes a substantial change of the RMS surface roughness, which settles to a RMS value of $R_q=7.2$ nm and an arithmetical deviation of $R_a=5.6$ nm. Such small surface bubbles are compatible in terms of average diameter with those observed by TEM in the $\hbox {TiO}_2$ capping-layer; in fact, the formation of bubbles in the capping-layer introduces a change that can be seen on the surface morphology.

**Figure 7:**
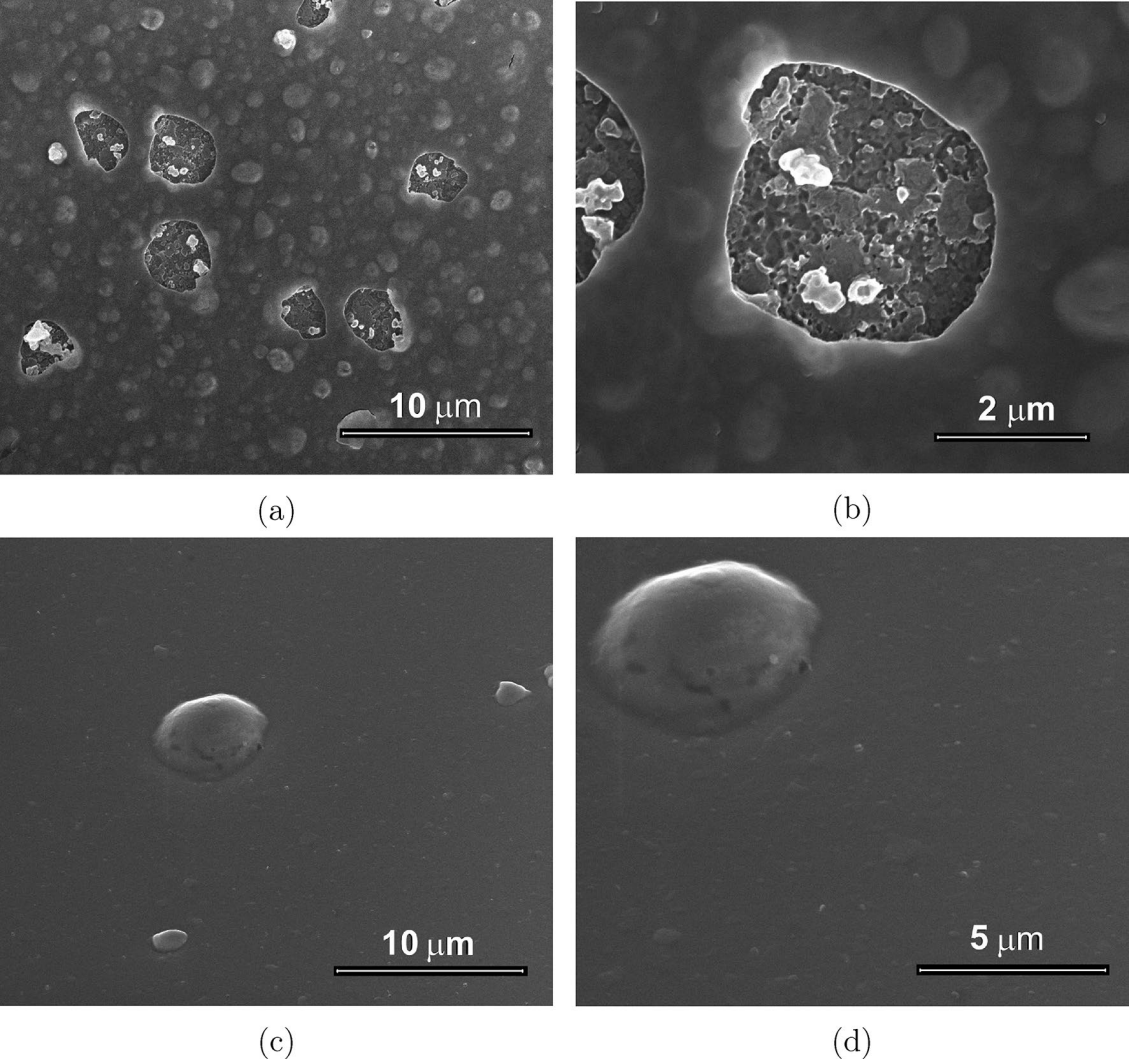
SEM images of the samples irradiated with 16 keV (**a**,**b**) and 100 keV (**c**,**d**) He ions.

In contrast, the 16 keV irradiated sample reveals a completely changed morphology, as shown by SEM analysis (Fig. 7a). The surface is rich in bubbles with areas of few $\mu$m in diameter (i.e. ranging from 1 to 3 μm), where the $\hbox {TiO}_2$ capping-layer appears delaminated (Fig. 7b). Such dramatic surface morphology change is also confirmed by a high value of RMS roughness ($R_q=83.6$ nm) and of arithmetical deviation ($R_a=60.8$ nm) measured by using AFM. These roughness values, which are much higher than those observed in the non-irradiated or in the 4 keV case, suggest that the dominant optical performance degradation observed for this sample is reasonably given by both surface and volume scattering. Moreover, AFM images show that the depth of these delaminations is about 90–150 nm (Fig. 6c), proving that for such areas the detachment involves the whole $\hbox {TiO}_2$ protective layer. The formation of such delaminations is directly connected to the bubble formation process occurring during irradiation. Since the implantation peak at 16 keV is placed exactly at the interface between Al and the $\hbox {TiO}_2$ protective layer, the accumulation of the implanted ions at this interface leads to the surface blistering, forming bubbles of various sizes; the formation of the bigger bubbles likely occurs through the fusion of smaller bubbles which migrate within the metal layer. In case a bubble swells too much, breakage can occur, resulting in the removal of the overlying protection layer. Focusing the attention on the delaminated areas having the smallest diameters (i.e. around 1 μm), in almost all cases, these can be directly attributed to a partial breakage of a bubble characterized by a height of at least 100 nm; typical examples are the structures marked by A, B, and D in Fig. 6c. The larger areas, like structure C in Fig. 6c, are instead due to a full breakage of a bubble, and they usually have a diameter between 3 and 5 μm. Thus, it is reasonable to assume that the diameter of bubbles having heights larger than 80–100 nm falls in the 3–5 μm range. This hypothesis is further strengthened by analyzing the number of bubbles per unit of surface area versus their diameter, considering only the structures higher than the sample $R_q$ value (see Fig. 6d). This analysis shows that the bubble population is characterized by a high density of sub-micron diameter bubbles, with the density rapidly decreasing for values of around 1.5 μm. Few bubbles with diameters larger than 2 μm have been observed, whilst bubbles with diameters larger than 3 μm are very rare. In fact, it can be concluded that the $\hbox {TiO}_2$ top-most layer is no longer homogeneous after the 16 keV-irradiation, due to delamination, blistering and inter-diffusion with the metal underneath. This conclusion perfectly matches the ellipsometric analysis previously discussed, in which a reasonable fitting of the experimental data can be carried out by only considering a very high surface roughness and a significant inter-diffusion between the dielectric and metal layers.

**Figure 8:**
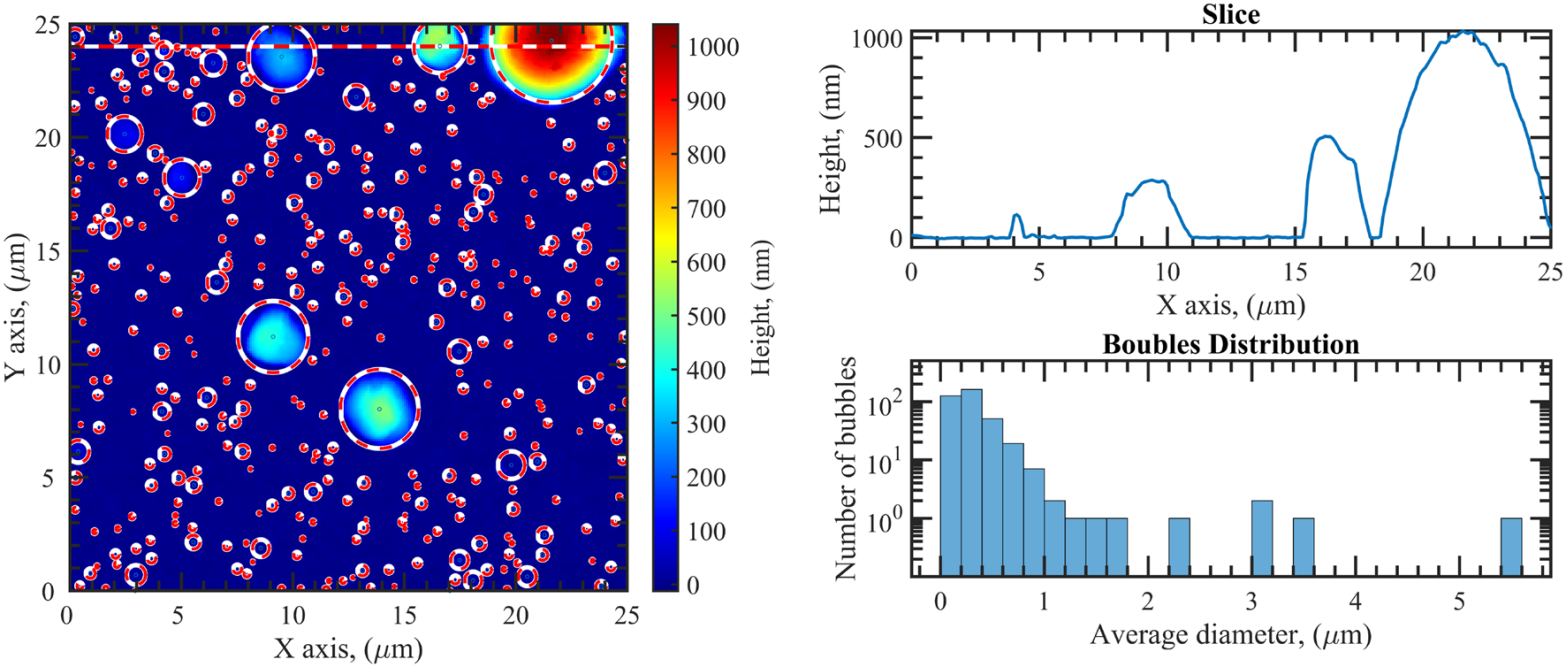
AFM image of the sample irradiated with 100 keV He ions (on the left). On the right: a height profile for the evaluation of the bubble sizes and the bubble count as function of their diameter.

In the case of the 100 keV-irradiated sample, SEM images show a surface populated with sub-micron bubbles which coexist with a few large bubbles with diameters ranging from 1 to 5 μm (Fig. 7c,d). AFM images confirm the presence of a large number of bubbles with a diameter smaller than 1 μm and a height of a few tens of nm (Fig. 8). Additionally, single bubbles with diameters larger than 1 μm and heights of hundreds of nm are observed. As shown in Fig. 1, the ion implantation peak falls within the substrate and the implanted ion density increases slowly throughout the Al film depth. Also in this case, bubbles forming on the metal layer can grow in size due to migration and diffusion phenomena with a consequent appearance of blistering on the surface. However, since at 100 keV the implanted ion concentration in the Al film is up to 100 times lower than that in the 16 keV case, the number of bubbles forming at the surface is limited and not sufficient for a huge morphological modification as observed in the 16 keV case. Furthermore, considering that at 100 keV the implanted ions density at the Al/substrate interface is about 30 times higher than that at the $\hbox {TiO}_2$/Al interface, bubble formation close to the interface with the substrate is higher, as supported by TEM analysis. Bubbles in the deep part of the Al film can grow, reaching considerable sizes since the portion of the film located above offers high resistance to breakage. The formation of such bubbles at the Al/substrate interface is directly responsible for the scattering observed on this sample.

## Conclusions

A $\hbox {TiO}_2$/Al bi-layer coating was irradiated with a He ion beam at three different energies to investigate the damage dependence on the ion energy. The design of the irradiation experiment was optimized in order to have the lower-energy 4 keV ions fully implanted into the $\hbox {TiO}_2$ capping-layer, the 16 keV ions implanted at the metal-dielectric interface, and the 100 keV ions implantation peak placed at the substrate interface. TEM analysis confirms the implantation profile as predicted. In the case of 4 keV ions, formation of small bubbles (about 10 nm in diameter) is proven to occur in the dielectric layer, which leads to an effective refractive index change. The bubbles do not move inside the dielectric and do not agglomerate, so that the morphology of the surface is preserved. In the 16 keV case, bubbles of different size form in the Al layer near the interface with the dielectric capping-layer, with the bigger ones likely to occur through the fusion of smaller bubbles which migrate inside the metal layer. A strong change of the surface morphology occurs, appearing highly blistered, and causing large scattering responsible for a specular reflectance drop. TEM analysis reveals that, due to bubble formation, the Al thickness increases by at least 25$\%$. In case bubbles swell too much (the limit in height has been found to be at least 100 nm), delamination occurs with a dramatic degradation of the top surface. Finally, in the 100 keV case, the implantation peak falls in the substrate, so that the concentration of implanted ions in the top part of the film structure is much lower than in the previous case. Only a few large bubbles with diameter greater than a micron are visible on the surface, since they mostly form at the interface between the metal and the substrate.

Our analysis demonstrates the importance of carefully selecting the irradiation parameters when testing materials and thin films’ radiation hardiness for application in space. The energy of the ions defines the region of implantation in the sample. Hence, by tuning it, it is possible to stress different depths of the component and, therefore to test the hardiness of different structural parts. The dose level, which in the present paper has been the same for all the investigated energies, also needs to be varied to reach the desired implantation density at the selected depth. In conclusion, in order to space-qualify an optical component, an analysis of the particles present in the mission radiation environment needs to be combined with the design of an experiment that takes the complexity of the component structure into account; only a fine tuning of the ion beam, which should be used as a probe, allows a full investigation of the potential degradation induced by the ion irradiation.

## Supplementary Information

Supplementary Information.
