# Supplementary material for: Dependence of the damage in optical metal/dielectric coatings on the energy of ions in irradiation experiments for space qualification
Source: Sci Rep. 2021 Feb 9;11:3429. doi: 10.1038/s41598-021-82860-7 (PMC7873038; doi:10.1038/s41598-021-82860-7)
Supplement: Supplementary file 1 — Supplementary Information. [file 41598_2021_82860_MOESM1_ESM.pdf]

Supplementary material

Maria G. Pelizzo[a,*], Alain J. Corso[a,+], Giovanni Santi[a,b], René Hübner[c], Denis Garoli[d,e], Dominic Doyle[f], Philip Lubin[g], Alexander N. Cohen[g], Jacob Erlikhman[g], Giulio Favaro[h], Marco Bazzan[h], Jon Drobny[i], Davide Curreli[i], and Maxim Umansky[j]

[a]*Consiglio Nazionale delle Ricerche - Istituto di Fotonica e Nanotecnologie (CNR-IFN), via Trasea, 7, 35131 Padova, Italy*

[b]*Università di Padova, Centro di Ateneo di Studi e Attività Spaziali (CISAS), via Venezia, 15, 35131 Padova, Italy*

[c]*Helmholtz-Zentrum Dresden-Rossendorf Institute of Ion Beam Physics and Materials Research Ion Beam Center Bautzner Landstr. 400, 01328 Dresden, Germany*

[d]*Istituto Italiano di Tecnologia – Via Morego, 30, I-16163 Genova, Italy*

[e]*Faculty of Science and Technology Free University of Bozen, Piazza Università 5, 39100 Bolzano, Italy*

[f]*ESTEC-European Space Agency, Keplerlaan 1, NL-2200 AG Noordwijk ZH, The Netherlands*

[g]*Department of Physics, University of California - Santa Barbara, CA, 93106*

[h]*Dipartimento di Fisica e Astronomia, Università di Padova, via Marzolo, 8, 35131 Padova*

[i]*Department of Nuclear, Plasma, and Radiological Engineering, University of Illinois at Urbana Champaign, Urbana, IL, 61801, USA*

[j]*Lawrence Livermore National Laboratory, Livermore, CA, 94550, USA*

Supporting note 1

Ellipsometric analysis

The experimental reflectance curves reported in the main text demonstrate that the He ions exposure modify the optical properties of the Al/TiO_2 bilayer. In order to better investigate these optical modifications, additional spectroscopic ellipsometry measurements have been performed (see Materials and Methods section). Ellipsometry is a powerful technique, which allows the retrieval of thin film parameters, such as the real and imaginary part of the optical constants, by modelling the experimental measurements.

Supporting Figures 1a and 1b report the obtained refractive index (real and imaginary parts) for TiO_2 layer in the four analyzed samples. As shown, the TiO_2 optical constants in the exposed samples change significantly; they were obtained by a proper fit of the experimental data using an appropriate model for each sample. The ellipsometric data from the as-deposited sample were fitted by using the dielectric constants available in the literature for both Al and TiO_2 [1, 2], by considering the nominal thicknesses of 340 and 90 nm for Al and TiO_2, and by setting a surface roughness with an R_q of 3.2 nm. Moreover, starting from such tabulated data (as reported in Figure 1), a model based on a dispersion law and/or oscillators was built for each material. TiO_2 was well described by using a simple Cauchy dispersion model with an Urbach absorption; instead, Al was described by combining a Drude and a Lorentz oscillator.

In the case of the sample exposed at 4 keV, a model considering only changes in the capping-layer, but not the Al layer, was considered, as this is not affected by the irradiation. Assuming nominal thicknesses and a surface roughness R_q of 6 nm (as retrieved by AFM), the ellipsometric curves can be properly fitted by varying only the TiO_2 optical constants. The optical constants of the TiO_2 in the 4 keV irradiated sample can still be described by a Cauchy dispersion law, as long as it is combined with two Lorentz oscillators which take into account the absorption coefficient induced by ion irradiation inside the capping-layer. On the contrary, the effect of He ion exposures at 16 and 100 keV is harder to model, since there are dramatic changes of the surface roughness and deep penetration of the ions in the Al layer. In the case of the 16 keV-irradiated sample, the fit was performed by a model with a high surface roughness and an inter-layer between the Al and TiO_2. Such an inter-layer is used to model the bubbles (voids) inside the Al, as revealed by

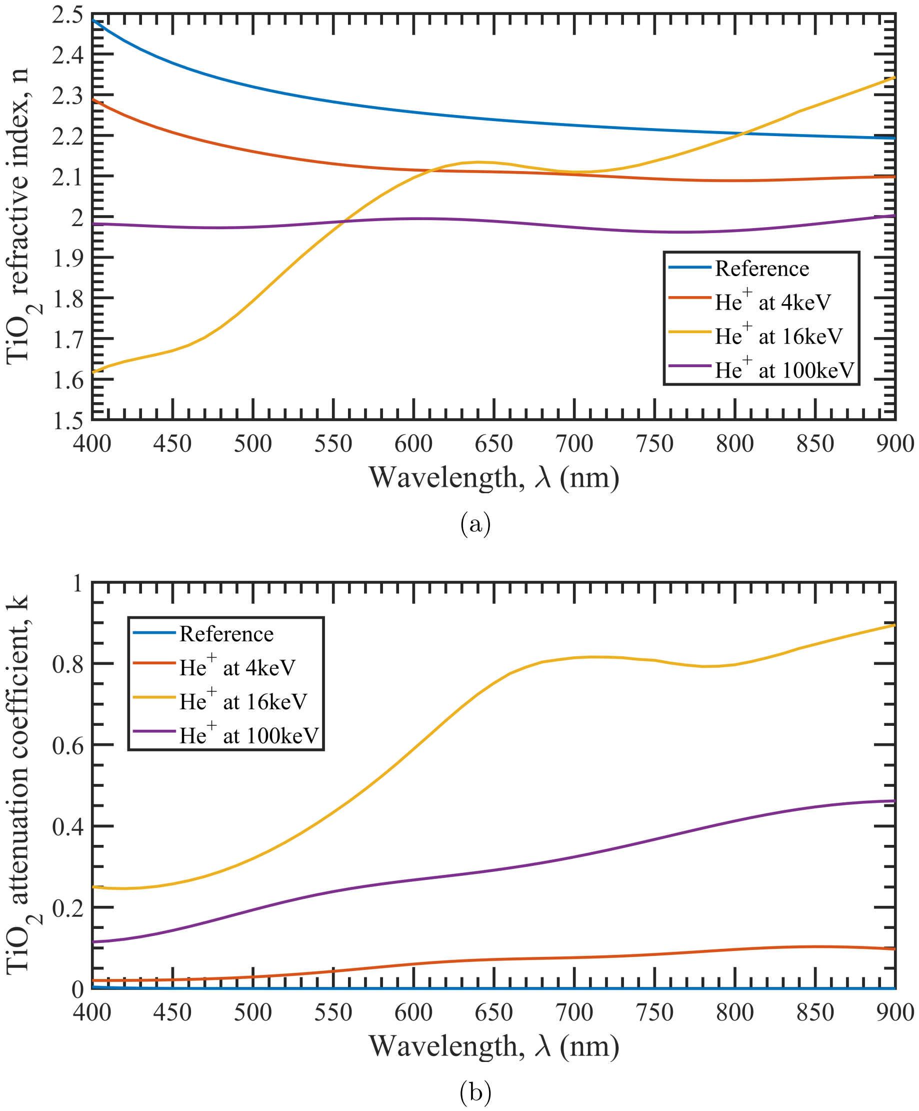

Figure 1: (a): Real part of the TiO_2 refractive index n for the reference and the irradiated samples. (b): Attenuation coefficient (imaginary part of the refractive index) k of TiO_2 layer for the reference and the irradiated samples.

TEM analysis (see Section 3.2 in the main text), by the use of a depth graded Effective Media Approximation (EMA). In the fitting process, a change of the Al optical constants was included with the constraint that the material is still described by a Drude and Lorentz oscillator-based model (see Figure 2). The best fit was obtained by using a surface roughness R_q of about 70 nm and a Maxwell-Garnet EMA model which assumed a volume fraction linearly scaled from 0.25 up to 0 with the depth. The thickness of this inter-layer was around 150 nm, a value perfectly compatible with those retrieved by TEM images. The fit included also a small modification of the Al optical constants, but a dramatic change for the TiO_2 that can be successfully modeled only by using a set of many oscillators. Thus, although this model is speculative in nature, it allows us to conclude that the irradiation at 16 keV has also deeply changed the TiO_2 capping-layer in term of its optical behaviour. Finally, the ellipsometric measurements of the sample irradiated with He^+ ions at 100 keV were fitted by using the same model employed for the 4 keV case, but by also changing the Al optical constants and setting a surface roughness R_q of about 18 nm, a value compatible with those retrieved by TIS analysis.

References

[1] Swagato Sarkar, Vaibhav Gupta, Mohit Kumar, Jonas Schubert, Patrick T. Probst, Joby Joseph, and Tobias A.F. König. Hybridized guided-mode resonances via colloidal plasmonic self-assembled grating. *ACS Applied Materials & Interfaces*, 11(14):13752–13760, 2019.

[2] Aleksandar D. Rakić. Algorithm for the determination of intrinsic optical constants of metal films: application to aluminum. *Applied Optics*, 34(22):4755–4767, Aug 1995.

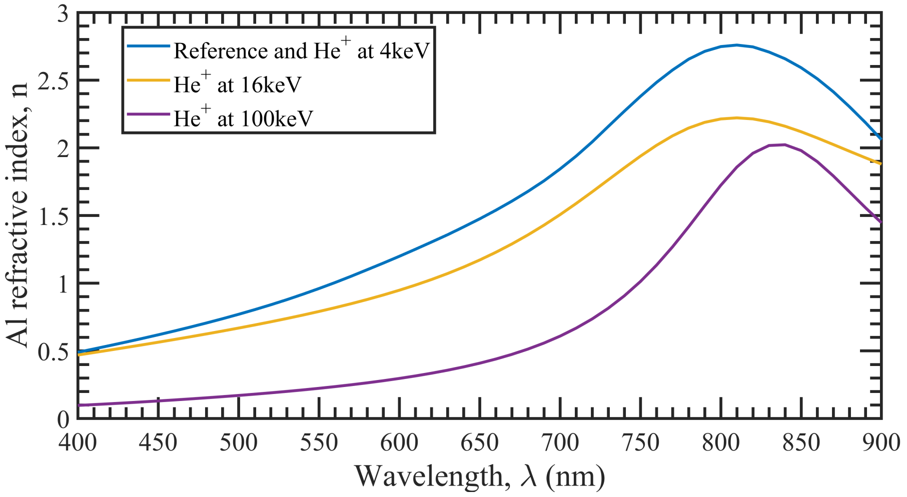

(a)

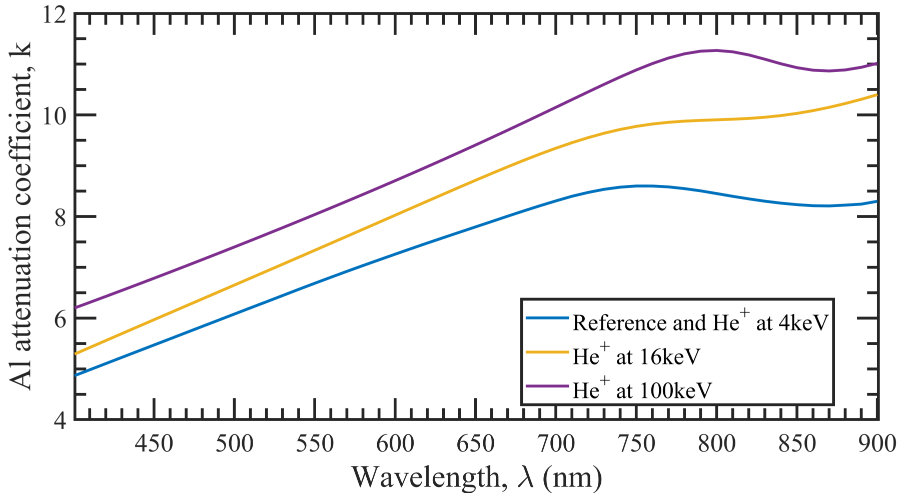

(b)

Figure 2: (a): Real part of the Al refractive index n for the reference and the irradiated samples. (b): Attenuation coefficient (imaginary part of the refractive index) k of Al layer for the reference and the irradiated samples.
